# The effects of duration of any breastfeeding on body mass index in Australian children: Exploration of health, economic and equity impacts

**DOI:** 10.1111/ijpo.13167

**Published:** 2024-08-29

**Authors:** Joseph Carrello, Vicki Brown, Anagha Killedar, Alison Hayes

**Affiliations:** ^1^ School of Public Health, Faculty of Medicine and Health The University of Sydney Sydney New South Wales Australia; ^2^ Deakin Health Economics, Institute for Health Transformation Deakin University Burwood Victoria Australia; ^3^ Menzies Centre for Health Policy and Economics, Faculty of Medicine and Health The University of Sydney Sydney New South Wales Australia

**Keywords:** BMI, breastfeeding, economic, equity, impacts

## Abstract

**Background:**

Breastfeeding is a protective measure against childhood overweight and obesity. However, many children are not breastfed the recommended duration, with those from disadvantaged backgrounds more likely to cease breastfeeding early.

**Objectives:**

Investigate the association between duration of any breastfeeding and body mass index (BMI) and estimate the health, economic and equity impacts of increasing breastfeeding duration to at least 6 months.

**Methods:**

We modelled the association between any breastfeeding duration and BMI at age 6/7 years, using a nationally representative cohort of 3935 Australian children (survey weighted to 221 103 children). We then used a simulation model to predict the impact of increasing breastfeeding duration to at least 6 months in all children on prevalence of overweight (including obesity) and associated healthcare costs to age 16/17 years.

**Results:**

Achieving breastfeeding duration of at least 6 months could prevent 2933 cases of overweight at age 16/17 years, translating to healthcare cost‐savings of AUD $4.29 million. Although most cases (68%) would come from low socio‐economic backgrounds this would make only a minor difference in reducing inequalities.

**Conclusion:**

Efforts to support increased breastfeeding duration could result in reduced prevalence of overweight and obesity and save healthcare costs, however, additional action would be required to improve equity.

## 1 INTRODUCTION

The benefits of breastfeeding on child health and development are widely recognized. Children who are not breastfed are more likely to suffer from diarrhoea,^1^ respiratory illness,^2^ type 1 diabetes^2^ and childhood leukaemia,^2^ and may have reduced cognitive development.^3^ Additionally, there is growing evidence that children who are not breastfed may face increased risk of overweight and obesity,^4^, ^5^ a serious public health issue in many nations including Australia, where one in four children are above a healthy weight.^6^

The World Health Organization (WHO) and UNICEF recommend early initiation of breastfeeding post birth, exclusive breastfeeding up to 6 months and continued breastfeeding alongside solid foods up to 2 years of age and beyond.^7^ These recommendations are reflected in the Australian Infant Feeding Guidelines, which also encourage exclusive breastfeeding up to 6 months and continuation of breastfeeding up to at least 1 year of age.^8^

While rates of breastfeeding initiation in Australia are high, it is estimated that only 60% of children still receive breastmilk by 6 months of age.^9^ This is a concern as it means many children may miss out on the full health benefits of breastfeeding, including prevention of overweight and obesity. Further, there is evidence that women from low socio‐economic backgrounds,^10^ with lower education levels,^10^ of Aboriginal and Torres Strait Islander status^11^ and living with overweight or obesity^10^ are most likely to cease breastfeeding early. Thus, failing to address breastfeeding duration has negative implications for health equity.

The WHO Report of the Commission on Ending Childhood Obesity made several recommendations to support breastfeeding as a contributor to improving early childhood nutrition and preventing overweight and obesity.^12^ This included promoting the benefits of breastfeeding for both the mother and child through broad‐based education to parents and the community at large, as well as regulatory measures to support breastfeeding, such as maternity leave, facilities and time for breastfeeding in the workplace.^12^ These recommendations are echoed by several national strategies in Australia, including the 2019 Australian National Breastfeeding Strategy,^13^ the National Women's Health Strategy 2020–2030^14^ and the National Obesity Strategy 2022–2032.^15^

Findings from Australian studies using data from national and state (Western Australia) cohorts of children support claims that breastfeeding for ≥6 months provides protective effects against later overweight and obesity.^16^, ^17^ Further, there is evidence that exclusive breastfeeding may offer greater protective effects than any breastfeeding.^17^, ^18^ While this suggests a dose–response relationship between breastfeeding duration and child body mass index (BMI) exists, the literature investigating this association is mixed. For example, a 2020 meta‐analysis found an inverse relationship between breastfeeding duration and child BMI at age 2–6 years,^19^ however, a later 2021 systematic review found insufficient evidence to determine the relationship between the duration of any breastfeeding (among breastfed infants) and overweight and obesity at age 2 years and beyond.^4^

Additionally, the longer‐term health, economic and equity benefits of increasing breastfeeding duration as a prevention measure against overweight and obesity are unknown. This information would be valuable to decision makers who need to weigh up investment in policies and programmes to support breastfeeding with competing demands for scarce resources.

As such, the aims of this study were to:

1. Investigate the association between duration of any breastfeeding and child BMI in a nationally representative longitudinal cohort of Australian children;
2. Use these results to estimate the potential health, economic and equity benefits (related to overweight and obesity prevention) which could be achieved from increasing breastfeeding duration to at least 6 months in all children in this cohort.

## 2 METHODS

### 2.1 Investigating the association between breastfeeding duration and early childhood body mass index

#### 2.1.1 Study sample and variables

The Longitudinal Study of Australian Children (LSAC) is a large, nationally representative study that has followed two cohorts of Australian children since 2004, a ‘B’ (infant) cohort and a ‘K’ (kindergarten) cohort (aged 0–1 years and 4–5 years at enrolment, respectively).^20^ Data collection has occurred in waves biennially, including direct measurements of height and weight for calculation of BMI based on WHO growth references.^21^

For the purposes of our study, we used data from 3935 children aged 6/7 years, sourced from wave 4 (calendar year 2010) of the ‘B’ cohort. The LSAC sampling design allows for valid inferences to be made about the entire national population of children in this age cohort using survey estimation techniques (*n* = 221 103).^22^

Child BMI at age 6/7 years was chosen as the dependent variable as this was the earliest age at which inequalities in BMI based on duration of any breastfeeding of 6 months or less were observed in our dataset (see Appendix 1 in Supplementary material). Duration of any breastfeeding (in days) was the primary independent variable, measured via parental response to the question ‘How old was the child when he/she completely stopped being breastfed?’ This was recorded at wave 2 (calendar year 2006), when study children were aged 2–3 years.

Socio‐economic position (SEP) was recorded as a *z*‐score that combined parents' education, occupation and family income.^23^ We categorized this variable into high and low SEP groups corresponding to a *z*‐score of above or equal to 0, and a *z*‐score below 0, respectively. Breastfeeding duration was analysed by these groups to identify socio‐economic disparities within the study population.

#### 2.1.2 Statistical analysis

Preliminary analysis revealed a non‐linear relationship between duration of any breastfeeding and child BMI at age 6/7 years (see Appendix 2 in Supplementary material). This was addressed by partitioning duration of any breastfeeding into two segments and conducting piecewise linear regression with survey estimation (*svy:regress* command in Stata).^24^ This method involves fitting separate regression lines before and after a defined ‘knot point’ in the primary independent variable. We trialled knot points at breastfeeding duration of 4, 5 and 6 months, with plots of modelled versus measured BMI assessed visually to identify the knot point of best fit.

Following this a multivariable piecewise linear regression model was fitted, adjusting for potential confounders including sex,^25^ SEP,^10^, ^26^ Aboriginal and Torres Strait Islander status,^11^, ^27^ culturally and linguistically diverse (CALD) status,^28^ maternal BMI (postpartum),^10^, ^29^ birth weight^29^ and gestational age.^30^ Paternal BMI, also associated with child BMI,^31^ was available in the LSAC dataset but could not be included in the multivariable model due to considerable missingness (~35%). Interaction terms between breastfeeding duration and SEP, CALD and Aboriginal/Torres Strait Islander status were included to test for potential effect modification. To determine the final model a backwards elimination method was used, with non‐significant interaction terms (*p* > 0.01) and covariates (*p* > 0.05) removed sequentially.

### 2.2 Overweight and obesity prevention benefits of increasing breastfeeding duration to at least 6 months

The coefficient for breastfeeding duration (reflecting mean reduction in BMI per extra day of breastfeeding) from the final model was used to estimate an individual‐level effect on BMI at age 6/7 years from extending breastfeeding duration to 6 months. This was done by creating a variable identifying the number of extra days of breastfeeding each child in the study population required to reach 6 months of breastfeeding (for children already breastfed for 6 months or greater this was zero, for those not breastfed at all this was 182 days), then multiplying this number by the coefficient for breastfeeding duration (see Appendix 3 in Supplementary material for further information).

The health, economic and equity impacts of increasing breastfeeding duration to at least 6 months was then explored using a validated micro‐simulation model that estimates BMI trajectories by SEP in Australian children and adolescents (the EQ‐EPOCH model).^32^ We created two cohorts—a control cohort (the study population of 6/7 year olds with no BMI effect applied) and a scenario cohort (with an individual‐level BMI reduction from increasing breastfeeding duration to 6 months, described above, applied once at baseline). These cohorts were simulated in the EQ‐EPOCH model over a 10‐year time‐horizon (until children reached 16/17 years).

For each cohort, simulated BMI was used to define weight status based on WHO BMI‐for‐age cut‐points (healthy weight and overweight/obesity [collectively referred to as overweight from here on]).^21^ Costs from a healthcare system perspective (valued in 2023 Australian dollars [AUD]) were calculated based on age, sex and weight status^33^ and assigned annually with a 5% discount rate applied (see Appendix 3 in Supplementary material for further details). Only direct healthcare costs were accounted for, which include hospital services (public and private inpatient services, public emergency department and public hospital outpatient clinics), primary healthcare services (general practitioner and allied health services, pharmaceuticals and dental) and referred medical services (specialist services, medical imaging and pathology). Indirect costs, such as lost wages, financial costs to households and unpaid care from households were not included.

Following simulation, population‐level weight category prevalence at age 16/17 years and total healthcare costs (over the 10‐year simulation) were summarized for each cohort. Cases of overweight avoided and associated cost‐savings from increasing breastfeeding duration to at least 6 months were calculated from the difference between these estimates.

#### 2.2.1 Exploring uncertainty

To explore uncertainty around our potential cost‐savings estimate, we conducted one‐way deterministic sensitivity analyses using 95% confidence intervals of the BMI effect size from increasing breastfeeding duration, 95% confidence intervals of the relative healthcare costs of overweight compared to healthy weight (see Appendix 4 in Supplementary material), as well as alternative discount rates (3% and 7%).

## 3 RESULTS

### 3.1 Study population and characteristics of early breastfeeding cessation

Characteristics of the study population of children aged 6/7 years (born in 2004) can be seen in Table 1. Children who were breastfed less than 6 months had higher mean BMI at age 6/7 years and were more likely to be from low SEP backgrounds, of Aboriginal and/or Torres Strait Islander descent and/or whose mothers had a higher BMI.

**TABLE 1:** Characteristics of study population by breastfeeding duration.

	Breastfed <6 months (population size = 106 488)	Breastfed ≥6 months (population size = 114 615)
Sex *n* (%)		
Male	54 610 (51)	57 892 (51)
Female	51 879 (49)	56 723 (49)
SEP *n* (%)		
Low SEP	69 805 (66)	47 961 (42)
High SEP	36 683 (34)	66 654 (58)
Aboriginal/Torres Strait Islander *n* (%)		
Yes	6771 (6)	3839 (3)
No	99 717 (94)	110 776 (97)
CALD *n* (%)		
Yes	20 682 (19)	22 568 (20)
No	85 806 (81)	92 047 (80)
Mean gestational age in weeks (95% CI)	38.94 (38.82, 39.05)	39.26 (39.17, 39.35)
Mean birth weight kg (95% CI)	3.33 (3.30, 3.36)	3.46 (3.43, 3.48)
Mean maternal BMI kg/m^2^ (95% CI)	26.78 (26.36, 27.21)	24.63 (24.36, 24.89)
Mean child BMI at age 6/7 years (95% CI)	16.70 (16.57, 16.83)	16.41 (16.31, 16.51)
Child weight status prevalence at age 6/7 years *n* (%)		
Healthy weight	71 828 (67)	84 509 (74)
Overweight/obesity	34 660 (33)	30 106 (26)

Abbreviations: BMI, body mass index; CALD, culturally and linguistically diverse; CI, confidence interval; SEP, socio‐economic position.

**FIGURE 1:**
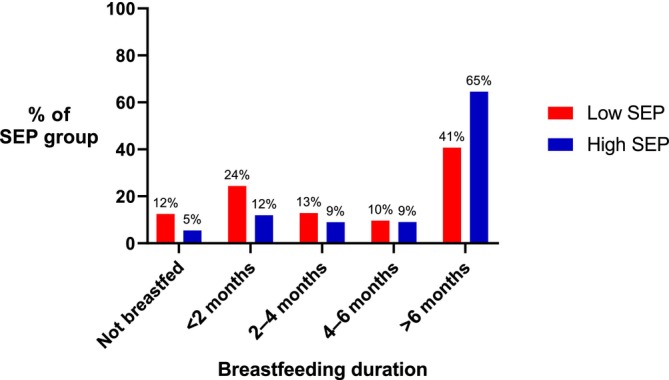
Breastfeeding duration by socio‐economic position.

Compared to children from high SEP backgrounds, those from low SEP backgrounds were more likely to have never been breastfed (12% vs. 5%) or breastfed only 1–2 months (24% vs. 12%) and less likely to be breastfed for 6 months or more (41% vs. 65%) (See Figure 1).

### 3.2 Association between breastfeeding duration and child BMI at age 6/7 years

For the simple piecewise linear regression model, a knot point at a breastfeeding duration of 6 months generated the model of best fit based on visual assessment (see Appendix 5 in Supplementary material). From the initial multivariable piecewise linear regression model, the coefficient for breastfeeding duration greater than 6 months was close to zero and not significant (*p* = 0.73), indicating no additional effect on child BMI at age 6/7 years. Subsequently, this coefficient was removed along with sex (*p* = 0.75) and interaction terms between breastfeeding duration and SEP, Aboriginal/Torres Strait Islander and CALD status, none of which were significant (*p* > 0.01).

Results of the final multivariable model can be seen in Table 2. There was evidence of a significant association between breastfeeding duration (up to 6 months) and BMI at age 6/7 years, after adjustment (*p* = 0.038). For each additional day a child was breastfed (up to 6 months), BMI at age 6/7 years was 0.0014 kg/m^2^ lower (or 0.043 kg/m^2^ lower per additional month). Assuming this association is causal, this means that a child who is not breastfed at all could have a reduction in BMI of up to 0.25 kg/m^2^ at age 6/7 years if they were breastfed up to 6 months (see Appendix 2 in Supplementary material).

**TABLE 2:** Multivariable piecewise linear regression results (*n* = 3290).

Variable	Coefficient (95% CI)	*p*‐value
Breastfeeding duration per additional day (up to 6 months)	−0.0014 (−0.0027, −0.000075)	0.038
Birth weight (per 100 g increase)	0.0006 (0.0004, 0.0008)	<0.001
Maternal BMI	0.09 (0.07, 0.11)	<0.001
SEP (high)	−0.19 (−0.37, −0.01)	0.038
Gestational age (weeks)	−0.074 (−0.12, −0.03)	0.003
Aboriginal and/or Torres Strait Islander (yes)	−0.55 (−0.92, −0.18)	0.004
CALD (yes)	0.35 (0.13, 0.56)	0.001
Constant	15.37 (13.70, 17.03)	<0.001

Abbreviations: BMI, body mass index; CALD, culturally and linguistically diverse; CI, confidence interval; SEP, socio‐economic position.

### 3.3 Overweight and obesity prevention benefits of increasing breastfeeding duration to at least 6 months

In our simulated population of 221 103 Australian children, increasing breastfeeding duration to 6 months in those children who experienced early cessation of breastfeeding (*n* = 106 488) was estimated to lead to 2933 cases of overweight avoided at age 16/17 years, the majority of which would be from children from low SEP backgrounds (*n* = 2004, 68%) (see Table 3).

**TABLE 3:** Prevalence of overweight and obesity by socio‐economic position at age 16/17 years under control and increased breastfeeding duration scenario conditions.

	Low SEP population (*n* = 117 767)	High SEP population (*n* = 103 336)	Total population (n = 221 103)
Control *n* (%)	43 646 (37%)	20 993 (20%)	64 639 (29%)
Scenario *n* (%)	41 642 (35%)	20 064 (19%)	61 706 (28%)
Difference *n* (%)	−2004 (−2%)	−929 (−1%)	−2933 (−1%)

**FIGURE 2:**
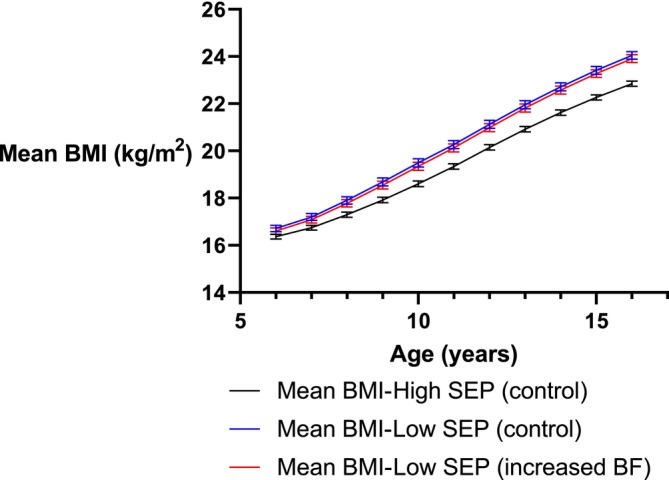
Mean body mass index trajectories by socio‐economic position.

However, this would make only a minor difference in reducing inequalities in the prevalence of overweight by SEP (from 17% to 16%). Even under a scenario of increased breastfeeding to 6 months through targeted action to low SEP groups only, the difference in prevalence of overweight at age 16/17 years would still be 15%. This can be seen visually in Figure 2; increased breastfeeding duration in low SEP children would result in only a minor reduction in mean BMI trajectories across childhood compared to children from high SEP backgrounds.

**FIGURE 3:**
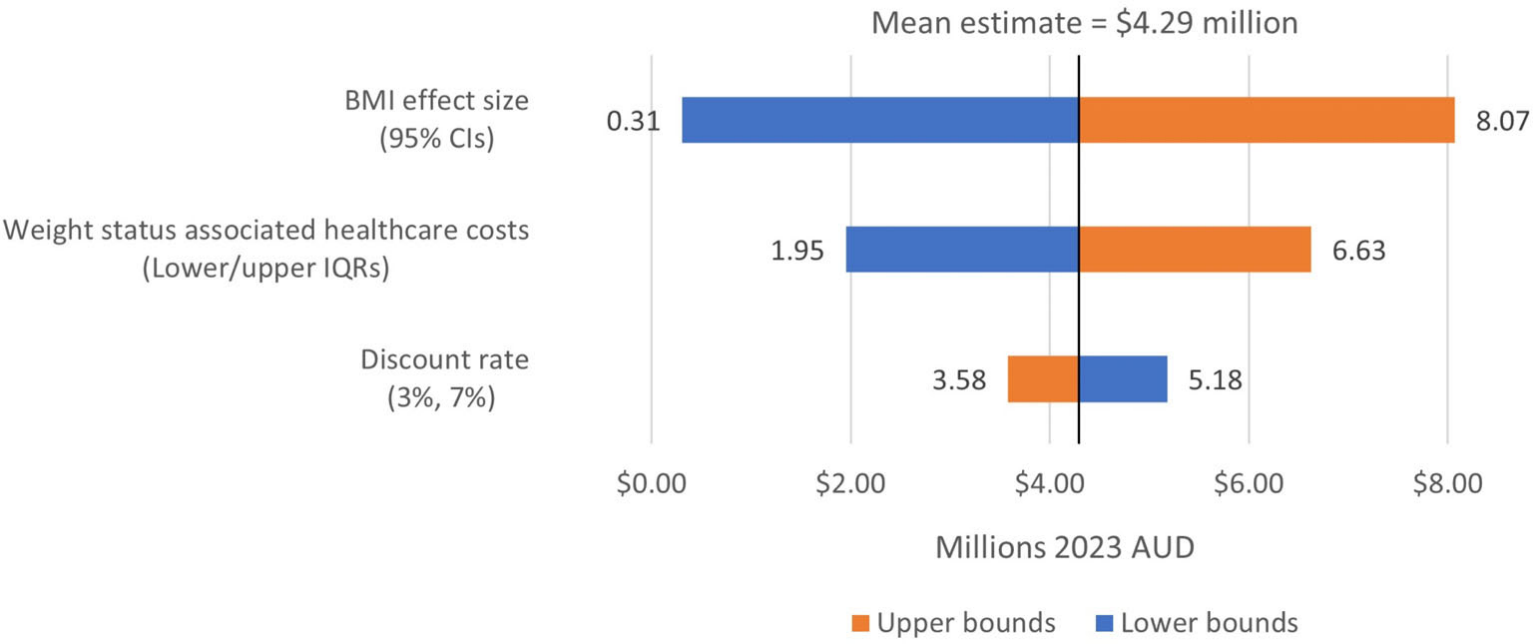
Sensitivity analyses of population cost‐savings estimates from increasing breastfeeding duration to 6 months.

The reduction in prevalence of overweight was estimated to translate to direct healthcare cost‐savings of $4.29 million AUD. However, this estimate is particularly sensitive to uncertainty around the BMI effect size from increasing breastfeeding duration, which could result in cost‐savings ranging from as little as $310 000 to as much as $8.07 million (See Figure 3).

## 4 DISCUSSION

In this study we have found evidence of an association between breastfeeding duration and a reduction in child BMI at age 6/7 years (−0.0014 kg/m^2^ per extra day of breastfeeding, up to 6 months of age). Under the assumption that this association is causal, we estimate that among a cohort of 221 103 Australian children born in one year, achieving a breastfeeding duration of at least 6 months in all children could potentially prevent 2933 cases of overweight at age 16/17 years and translate to direct healthcare cost‐savings of approximately $4.29 million (2023 AUD). As these are the benefits for a single annual cohort of births, the change in breastfeeding duration could potentially generate that benefit year after year. Although most cases of overweight prevented are estimated to come from low SEP backgrounds (*n* = 2004, 68%), this would make only a minor difference in reducing inequalities in the prevalence of overweight between low and high SEP groups, indicating more action than just targeted breastfeeding interventions is required to achieve weight status equity. It is important to note that our findings reflect a best‐case scenario where all children born in 1 year are breastfed to 6 months, which is unlikely to be achieved in practice.

The dose–response relationship between breastfeeding duration and reduced child BMI found in this study is consistent with previous systematic reviews and meta‐analyses which estimated that lowest odds of developing childhood obesity were associated with breastfeeding duration ≥6 and ≥7 months respectively.^19^, ^34^ Further, an earlier systematic review and meta‐analysis estimated that this association lasts up to a breastfeeding duration of 9 months.^35^ This is longer than the plateau in BMI effect beyond 6 months of breastfeeding found in our study, which may be attributable to differences in study populations and confounding factors.^35^

While previous studies have investigated the economic benefits of increasing breastfeeding rates, including in Australia,^36^, ^37^ they have tended to focus on a limited number of child illnesses, with only one US study including effects on obesity prevention in their estimates.^38^ Therefore, to the best of our knowledge, our study is the first to identify the specific economic benefits of increasing breastfeeding duration as an obesity prevention measure.

The socio‐economic disparities in breastfeeding duration found in our study are consistent with previous studies in Australia^10^, ^39^, ^40^, ^41^ and internationally.^42^ Recognized barriers to breastfeeding include societal pressures such as negative attitudes to breastfeeding in public, lack of access to breastfeeding education and professional lactation support, less supportive employment arrangements or workplace settings, religious and cultural beliefs and mental health issues.^13^ Maternity care practices should reflect the WHO Ten Steps to Successful Breastfeeding^43^ and the design of government policies and health promotion programmes must address these broader factors to improve the capacity of the most disadvantaged women to consider breastfeeding longer.^44^ However, given only the minor impact of increasing breastfeeding duration on reducing weight status inequalities, this must be in addition to other targeted childhood obesity prevention strategies to achieve equity.

Strengths of our study include the use of a large, nationally representative cohort of Australian children with individual‐level data, both to explore the association between breastfeeding duration and BMI and also as an input population for our modelled health, economic and equity estimates. Further, we were able to adjust for a large range of confounders in our statistical analysis and include breastfeeding duration as a continuous rather than categorical variable, which provides a more precise estimate of the magnitude of effect. The use of the EQ‐EPOCH microsimulation model to estimate SEP‐dependent BMI‐growth trajectories is another strength, providing valuable evidence of the potential health equity impacts from increasing breastfeeding duration.

A limitation of this study is that the measure of breastfeeding duration captured by the LSAC may be subject to error due to recall and social desirability bias. Additionally, it does not reflect duration of exclusive breastfeeding, which has evidence of greater protective effects against overweight and obesity compared to any breastfeeding.^17^, ^18^ Further, other dimensions of infant feeding related to breastfeeding practices and risk of developing overweight and obesity (such as early complementary feeding, knowledge and attitudes towards breastfeeding from parents and local food environments) were not accounted for in our analysis. All these factors have potentially influenced the extent of the relationship between breastfeeding duration and child BMI presented in this study.

The estimated health and economic benefits of increasing breastfeeding duration reported in our study are limited to those related to childhood overweight and obesity only. Other benefits from increased breastfeeding duration, such as child's cognitive development,^45^ reduced chronic disease risk in later‐life,^46^ related maternal health benefits such as reduced risk of cardiovascular disease, diabetes and breast cancer,^47^, ^48^, ^49^ or wider economic benefits (such as reductions in costs for families for infant formula)^50^ were not included. Consideration of these broader impacts of breastfeeding would likely increase the potential economic benefits that could be achieved through increasing breastfeeding duration.

In conclusion, increasing breastfeeding duration to at least 6 months in children experiencing early cessation of breastfeeding has the potential to generate health and economic benefits through effects on overweight and obesity prevention. However, despite a socio‐economic pattern in early breastfeeding cessation, achieving such a scenario is estimated to make only a minor impact in reducing inequalities in the prevalence of overweight and obesity. These findings will be useful to policymakers, those involved in infant health promotion and researchers in the design and implementation of early childhood obesity prevention interventions that incorporate breastfeeding components.

## AUTHOR CONTRIBUTIONS

JC, VB and AH conceived the study design. JC, AK and AH analysed data. All authors were involved in writing the paper and have final approval of the submitted and published versions.

## CONFLICT OF INTEREST STATEMENT

The authors declare no conflicts of interest.

## Supporting information


**Data S1:** Supporting information.
