# Supplementary material for: The effects of duration of any breastfeeding on body mass index in Australian children: Exploration of health, economic and equity impacts
Source: Pediatr Obes. 2024 Aug 29;20(2):e13167. doi: 10.1111/ijpo.13167 (PMC11710949; doi:10.1111/ijpo.13167)
Supplement: Supplementary file 1 — Data S1: Supporting information. [file IJPO-20-e13167-s001.pdf]

The effects of duration of any breastfeeding on body mass index in Australian children: exploration of health, economic and equity impacts

Joseph Carrello[1], Victoria Brown[2], Anagha Killedar[3], Alison Hayes[1]

[1] School of Public Health, Faculty of Medicine and Health, The University of Sydney

[2] School of Health & Social Development, Faculty of Health, Deakin University

[3] Menzies Centre for Health Policy and Economics, Faculty of Medicine and Health, The University of Sydney

Corresponding author: Joseph Carrello, email: jcar6556@uni.sydney.edu.au

Appendix 1.

Figure S1. Emergence of inequalities in child BMI based on duration of any breastfeeding <6 months and >6 months respectively

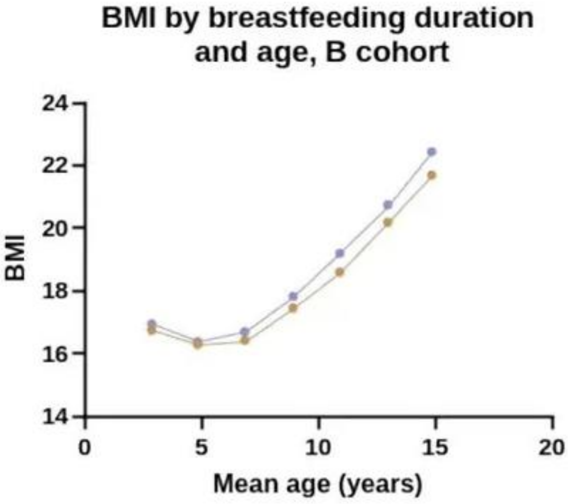

Appendix 2.

Figure S2. Non-linear relationship between breastfeeding duration and child BMI at age 6/7 years

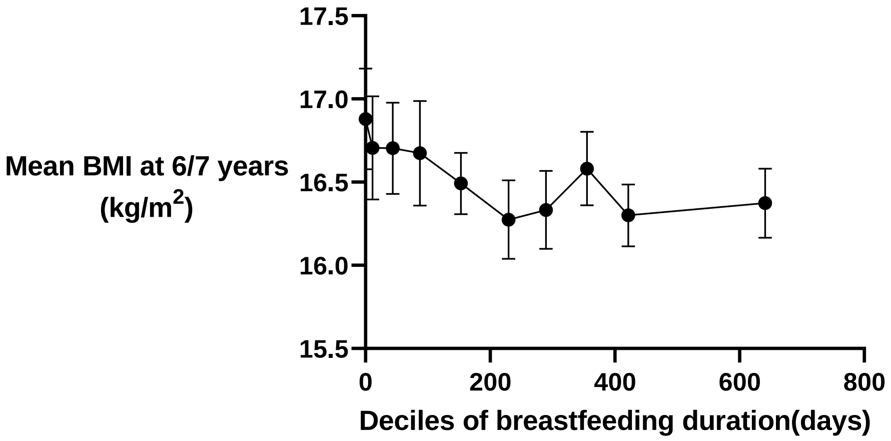

Appendix 3.

Table S1. Estimated BMI reduction at age 6/7 years from increasing breastfeeding duration to 6 months

Original breastfeeding duration of child	Number of children in study population n (%) [a]	Mean (95% CI) reduction in BMI at age 6/7 years
Not breastfed	20,285 (9)	-0.25 kg/m^2 (-0.01, -0.49 kg/m^2)
Breastfed 1 month	29,467 (13)	-0.21 kg/ m^2 (-0.01, -0.41 kg/m^2)
Breastfed 2 months	11,564 (5)	-0.17 kg/ m^2 (-0.009, -0.33 kg/m^2)
Breastfed 3 months	23,298 (11)	-0.13 kg/ m^2 (-0.007, -0.25 kg/m^2)
Breastfed 4 months	11,114 (5)	-0.08 kg/ m^2 (-0.005, -0.16 kg/m^2)
Breastfed 5 months	10,760 (5)	-0.04 kg/ m^2 (-0.002, -0.08 kg/m^2)
Breastfed 6 or more months	114,615 (52)	0 kg/ m^2

[a] Note that Breastfeeding duration was measured in days-numbers provided in table are rounded to nearest month

Appendix 4. Details of direct healthcare costs used in EQ-EPOCH model

Annual healthcare costs were calculated based on age, sex and weight status using a 'top-down approach', using methods previously described.[1] Mean annual healthcare costs by age group and sex were sourced from national administrative records of health expenditure in Australia 2018–19 and include hospital services (public and private inpatient services, public emergency department and public hospital outpatient clinics), primary healthcare services (general practitioner and allied health services, pharmaceuticals and dental) and referred medical services (specialist services, medical imaging and pathology).[2] All healthcare costs were inflated to 2023 AUD using the Australian Institute of Health and Welfare (AIHW) total health price index.[3] The prevalence of healthy weight (including underweight) and overweight (including obesity) by age group and sex was sourced from the 2017-18 Australian National Health Survey.[4] Using this data and the relative cost of overweight (including obesity) compared to healthy weight of 1.129 (13%) reported in Au, et al [5] we then calculated the average annual healthcare cost per child by age group, sex and weight status. For sensitivity analyses, we modelled low and high estimates of healthcare costs for children with overweight (including obesity), derived from the lower and upper confidence bounds of the relative cost of overweight (including obesity) compared to healthy weight [6] of 6% and 20% reported in Au, et al. [5]

Table S2. Annual healthcare costs attributed to children in the EQ-EPOCH model

Age group (years)	Mean annual healthcare costs	Prevalence Healthy weight (including underweight)	Prevalence Overweight (including obesity)	Relative cost of overweight (including obesity) compared to healthy weight (95% CIs)	Mean annual healthcare costs- Healthy weight (including underweight)	Mean annual healthcare costs- Overweight (including obesity) (low, high)
**Boys**						
5-9	$1,521	0.75	0.25	1.13 (1.06, 1.20)	$1477	$1,668 ($1,564, $1,772)
10-14	$1,556	0.78	0.22	1.13 (1.06, 1.20)	$1,519	$1,715 ($1,608, $1,822)
15-19	$1,834	0.63	0.37	1.13 (1.06, 1.20)	$1,745	$1,971 ($1,848, $2,093)
**Girls**						
5-9	$1,234	0.76	0.24	1.13 (1.06, 1.20)	$1,192	$1,346 ($1,262, $1,429)
10-14	$1,454	0.77	0.23	1.13 (1.06, 1.20)	$1,415	$1,598 ($1,498, $1,698)
15-19	$2,548	0.71	0.29	1.13 (1.06, 1.20)	$2,450	$2,766 ($2,594, $2,939)

Appendix 5.

Figure S3. Piecewise linear regression model fit, using knot point at 6 months Breastfeeding

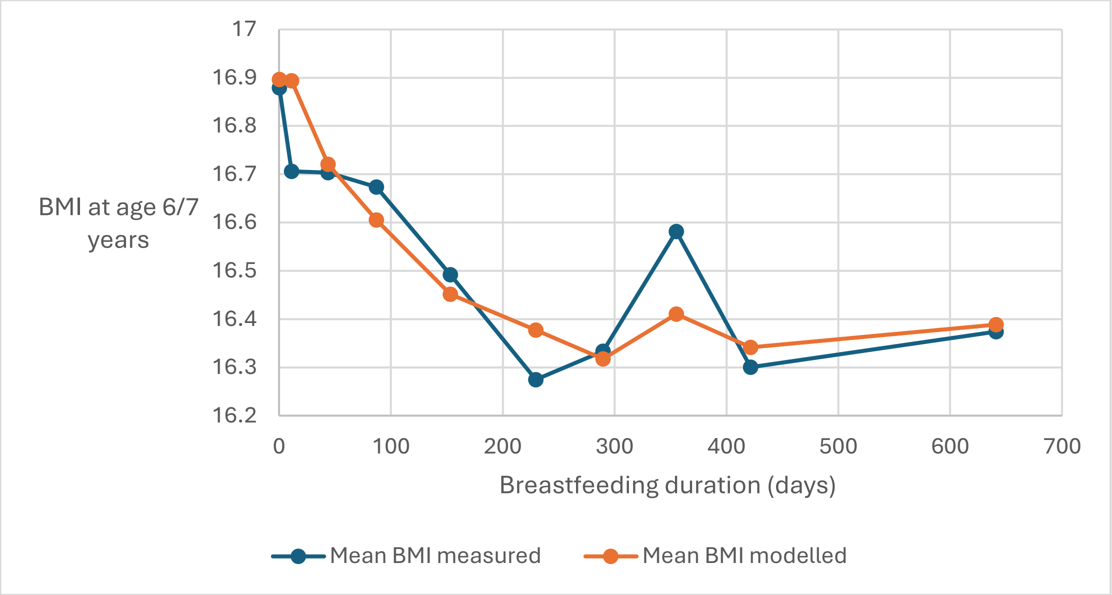

References

1. Killedar A, Lung T, Taylor RW, Taylor BJ, Hayes A. Is the cost-effectiveness of an early-childhood sleep intervention to prevent obesity affected by socioeconomic position? *Obesity*. 2023;31(1):192-202. doi:10.1002/oby.23592

2. Australian Institute of Health and Welfare. Disease expenditure in Australia 2018-19, Summary. Published August 25, 2021. Accessed March 3, 2023. https://www.aihw.gov.au/reports/health-welfare-expenditure/disease-expenditure-australia/contents/summary

3. Health expenditure Australia 2020-21, Concepts and definitions. Australian Institute of Health and Welfare. Published November 23, 2022. Accessed October 22, 2023. https://www.aihw.gov.au/reports/health-welfare-expenditure/health-expenditure-australia-2020-21/contents/overview-of-data-sources-and-methodology/concepts-and-definitions

4. Australian Institute of Health and Welfare. Overweight and obesity among Australian children and adolescents. Australian Institute of Health and Welfare. Published August 13, 2020. Accessed May 30, 2024. https://www.aihw.gov.au/reports/overweight-obesity/overweight-obesity-australian-children-adolescents/summary

5. Au N. The Health Care Cost Implications of Overweight and Obesity during Childhood. *Health Services Research*. 2012;47(2):655-676. doi:10.1111/j.1475-6773.2011.01326.x
